# Aquaporin-1 and Aquaporin-4 Expression in Ependyma, Choroid Plexus and Surrounding Transition Zones in the Human Brain

**DOI:** 10.3390/biom13020212

**Published:** 2023-01-22

**Authors:** Ronja Bihlmaier, Felix Deffner, Ulrich Mattheus, Peter H. Neckel, Bernhard Hirt, Andreas F. Mack

**Affiliations:** Institute of Clinical Anatomy and Cell Analysis, University of Tübingen, 72074 Tübingen, Germany

**Keywords:** aquaporin-1, aquaporin-4, choroid plexus, glymphatic pathway, water homeostasis, cerebrospinal fluid, blood–CSF barrier

## Abstract

The choroid plexus (CP) is a structure in the brain ventricles that produces the main part of the cerebrospinal fluid (CSF). It is covered with specialized cells which show epithelial characteristics and are the site of the blood–CSF barrier. These cells form a contiguous cell sheet with ventricle-lining ependymal cells which are known to express aquaporin-4 (AQP4). In contrast, CP epithelial cells express aquaporin-1 (AQP1) apically. We investigated the expression patterns of aquaporins in the CP-ependyma transition from human body donors using immunofluorescence and electron microscopy. Ependymal cells and subependymal astrocytes at the base of the CP showed a particularly high AQP4 immunoreactivity. Astrocytic processes formed a dense meshwork or glial plate around the blood vessels entering the CP. Interestingly, some of these astrocytic processes were in direct contact with the CP stroma, which contains fenestrated blood vessels, separated only by a basal lamina. Electron microscopy confirmed the continuity of the subastrocytic basal lamina with the CP epithelium. We also probed for components of the AQP4 anchoring dystrophin–dystroglycan complex. Immunolabeling for dystrophin and AQP4 showed an overlapping staining pattern in the glial plate but not in previously reported AQP4-positive CP epithelial cells. In contrast, dystroglycan expression was associated with laminin staining in the glial plate and the CP epithelium. This suggests different mechanisms for AQP4 anchoring in the cell membrane. The high AQP4 density in the connecting glial plate might facilitate the transport of water in and out of the CP stroma and could possibly serve as a drainage and clearing pathway for metabolites.

## 1. Introduction

After the discovery of water channels [1], aquaporins in the brain were detected in the mid-1990s [2,3,4]. In the central nervous system (CNS), aquaporin-4 (AQP4) is the most dominant water channel and is even considered to be one of the most abundant proteins in the brain [5]. It is expressed in all vertebrate groups in cells of the astroglial family, which includes astrocytes, radial glia and ependymal cells. In most astrocytes, the distribution of AQP4 is highly polarized, i.e., as revealed by immunohistological stains, there is a high density of this water channel in astrocytic membranes facing the basal lamina of blood vessels and the pia mater. In 1998, Rash et al. discovered that the so-called orthogonal arrays of particles (OAPs) seen in freeze–fracture preparations for electron microscopy could be labelled with AQP4 antibodies [6]. Until then, these OAPs were considered markers for astrocytic membranes in freeze–fracture replicas [7,8,9]. In that sense, the distribution of AQP4 in the form of OAPs had been investigated prior to the discovery and knowledge of aquaporins.

In their original paper describing CHIP (aquaporin-1) distribution, Nielsen, Smith, Christensen and Agre [4] localized this water channel to the apical membrane domain of rat choroid plexus (CP) epithelium cells, and subsequently a weak basolateral expression was also reported [10]. In contrast, ependymal cells lining the ventricles were positive for AQP4, and vice versa, AQP4 was not found to be expressed by the epithelial cells of the CP and ependymal cells were devoid of aquaporin-1 (AQP1) [11]. Functionally, the mostly apically localized AQP1 in CP epithelial cells is thought to facilitate cerebrospinal fluid (CSF) production, and expression of AQP4 by astrocytes has been implicated in brain water homeostasis [12,13,14].

Ventricle-lining ependymal cells form a continuous cell sheet with CP epithelial cells, yet differ in other properties other than the expression of AQPs, e.g., in the formation of tight junctions and the expression of transport proteins. Some cells in the transition zone from ependyma to CP have been reported to express AQP1 and AQP4 in the mouse brain (see Figure 5 in [14]), but no study has addressed the AQP expression in the CP attachment region of the human brain. Recently, we discovered that the strict, regionalized expression of AQP1 and AQP4 mentioned above does not hold for the human CP, where we found many AQP4-positive plexus epithelial cells with an irregular pattern, at least in the tissue of aged human donors [15].

Therefore, we investigated the transition zone between the ependyma and the CP epithelium more closely in human brain samples, including a peculiar glial structure forming the attachment of the CP to the brain parenchyma and surrounding the blood-supplying vessels, often referred to as the *Taenia choroidea*.

The fact that this glial plate covers the blood vessels enclosed by meningeal tissue, which extends into the CP stroma, and the fact that capillaries in the CP stroma are fenestrated raise the question of water flow through these structures. We report here that, other than in CP epithelial cells, there is a strong AQP4 immunoreactivity in the glial plate of the *Taenia choroidea* formed by the processes of astroglial cells. The extent of these astroglial processes in the stroma of the CP and their high density of water channels might have implications for water flow and homeostasis, at least in the aging human brain.

## 2. Materials and Methods

### 2.1. Human Post-Mortem Specimens

Human brain tissue was collected from seven individuals who volunteered to donate their bodies to the Institute of Clinical Anatomy and Cell Analysis in Tübingen. In concordance with the declaration of Helsinki, the donors gave their informed consent to the use of their cadavers for research purposes. The Ethics Committee of the Medical Department of the Eberhard Karls University in Tübingen approved the accuracy of this procedure under the project number 237/2007BO1. The post-mortem time of the tissue extraction did not exceed a time frame over 20 h according to the official death certificate provided by a medical professional. Details on the body donors are provided in Table S1.

### 2.2. Immunohistochemistry

The *Plexus choroidei* of the lateral ventricles were removed from the brains together with attached ependymal and subependymal tissue and fixed in 4% paraformaldehyde for 24 h. After being rinsed in PBS, the samples were further dissected and placed in 30% (*w*/*v*) sucrose for another 24 h for cryoprotection. The fixed samples were frozen in isopentane-nitrogen-cooled TissueTek^®^ (Sakura, Staufen, Germany) and stored at −80 °C before being cryosectioned at 18 μm.

Next, cryosections were rehydrated and washed in PBS for 10 min before being incubated in blocking solution containing PBS, 4% (*v*/*v*) goat serum (Biochrom, Berlin, Germany), 0.1% (*v*/*v*) bovine serum albumin (Roth, Karlsruhe, Germany) and 0.1% (*v*/*v*) Triton^®^ X-100 (Roth, Karlsruhe, Germany) for 90 min at room temperature.

Afterwards, sections were incubated with primary antibodies (Table 1) diluted in the preincubation solution overnight at 4 °C in a humidified chamber. After washing with PBS three times for 10 min, the secondary antibodies (Table 1) were applied for 90 min at room temperature. Afterwards, sections were stained with the nuclear stains DRAQ5 (1:1000; Thermo Fisher, Waltham, MA, USA) or DAPI (1:1000) and washed with PBS three times for 10 min before being mounted with Mowiol 4–88 (Roth).

### 2.3. Light Microscopy

Stained cryosections were analyzed, and images were scanned on a Zeiss LSM510 Meta confocal microscope (Zeiss, Oberkochen, Germany) equipped with an argon laser with an excitation wavelength at 488 nm and two helium–neon lasers with wavelengths for excitation at 543 nm and 633 nm, respectively, and appropriate filter sets. Some confocal images were acquired with an LSM900 Airyscan confocal system (Zeiss). Alternatively, for overviews, images were taken on an Axio Imager Z1 fluorescence microscope (Zeiss) with an Apotome module. The systems’ software, ZEN Black and Blue (Zeiss), were used for image acquisition, and image plates were assembled and processed with Adobe Photoshop CS2 (San José, CA, USA).

### 2.4. Electron Microscopy

Dissected pieces of CP tissue were immediately fixed in 2.5% glutaraldehyde buffered in 0.1 M cacodylate (pH 7.4) for 2 h. Samples were post-fixed in 1% osmium tetroxide in PBS (pH 7.4) for 1 h, subsequently dehydrated in a graded ethanol series and acetone and embedded in epoxy resin (Sigma Aldrich, Darmstadt, Germany). Semithin sections (1.5 µm) were taken and stained with methylene blue to identify regions of interest (i.e., the ependymal–CP transition area) before cutting ultrathin sections (50 nm). Ultrathin sections were analyzed, and images were recorded on a LEO 912AB transmission electron microscope (Zeiss, Oberkochen, Germany).

## 3. Results

**Figure 1:**
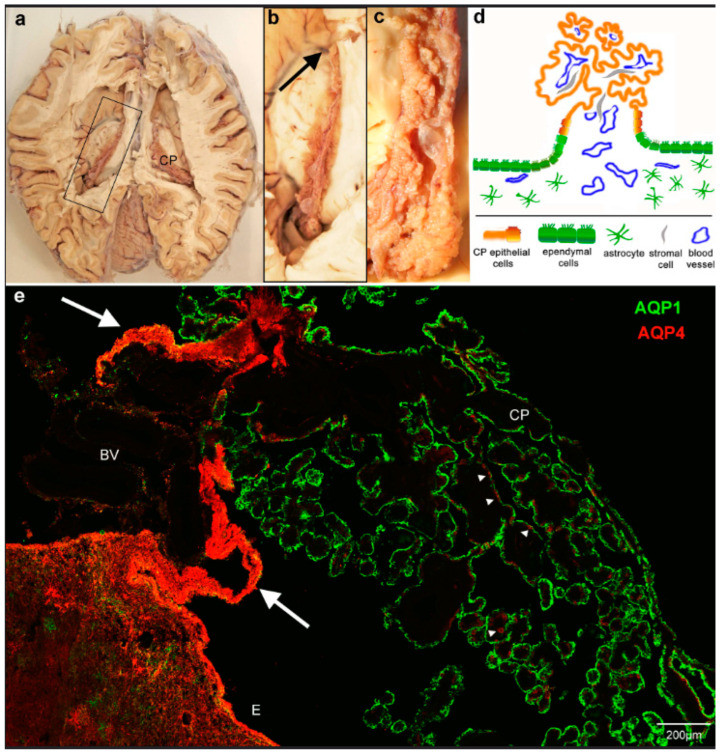
Overview of the location and attachment of the choroid plexus and aquaporin expression. (**a**) Horizontal section through a human brain with open lateral ventricles. (**b**) View of the boxed area indicated in a. showing the choroid plexus (CP) from the anterior end close to the interventricular foramen (arrow) to the most posterior part, where it thickens to the glomus and extends further into the lower horn of the lateral ventricle. (**c**) Detailed view of the posterior region shows the firm attachment to the thalamic surface. (**d**) The schematic diagram of a cross-section perpendicular to the plane shown in c indicates the direct transition of the ependyma to the choroid plexus epithelium. (**e**) Cryostat section through the choroid plexus and ependymal attachment of the lateral ventricle stained for aquaporin 1 and 4 (AQP1, AQP4). CP epithelium is positive for AQP1, astrocytic endfeet in the brain parenchyma and ependyma (E), and some cells (arrowheads) in the CP are positive for AQP4. Note that the transitional ependyma connecting to the CP epithelium and underlying tissue is strongly immunofluorescent for AQP4 (arrows). BV, blood vessels.

The human choroid plexus of the lateral ventricles is a prominent folded structure with protruding villi extending the entire length of the main ventricular cavity and further into the inferior (temporal) horn (Figure 1a–c). The outer surface is formed by a single-layered epithelium covering a stromal layer that contains blood vessels and connective tissue cells. The blood supply from the anterior and posterior choroidal arteries branches several times before entering the CP. The anatomical transition zone between the CP tissue and the thalamic tissue contains branches of these blood vessels and is referred to as *Taenia choroidea*. The CP epithelial layer forms a cell sheet that is continuous with the ependymal lining of the ventricles (Figure 1d). Focusing on the thalamic region and staining with antibodies against AQP4 and AQP1, we first confirmed the known pattern of apical AQP1 immunoreactivity in the CP epithelium and AQP4 reactivity in astrocytic endfeet and basolateral ependymal membranes, as well as the recently reported occurrence of AQP4 in some CP epithelial cells (Figure 1). AQP1 and AQP4 can be expressed in the same CP cells but are not colocalized intracellularly. In addition, sections through the CP and its attachment to the thalamus surface unexpectedly revealed many heavily AQP4-labeled cell processes underneath the epithelium and extending into the plexus stroma (Figure 1e). We verified this AQP4 staining pattern in the ependyma–CP transition of the lateral ventricles from six body donors. Throughout all our samples, the intensity of AQP4 on astrocytes in the glial attachment plate was stronger than on the astrocytes in the adjacent brain tissue.

**Figure 2:**
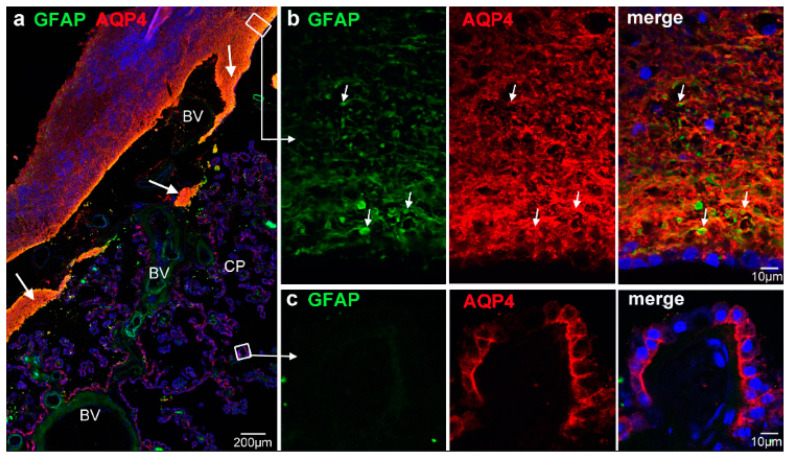
Subependymal tissue in the transitional zone is formed by astrocytes. (**a**) At low magnification, GFAP immunoreactivity largely overlaps with AQP4 stain under the transitional ependyma (large arrows). (**b**). Detailed view from the ependymal/subependymal region indicated by the white box shows a dense meshwork of GFAP-positive processes and strong AQP4 immunoreactivity. The GFAP processes are often surrounded or associated with AQP4 staining (small arrows). The surface ependymal cells are only weakly positive for GFAP. (**c**) GFAP stain is completely lacking in AQP4-positive CP epithelial cells. Nuclei are stained with DRAQ5. BV, blood vessels.

Secondly, we stained AQP4 in combination with GFAP in order to test whether the intensely labeled AQP4 processes were indeed astrocytic. There was an extensive overlap of GFAP and AQP4 reactivity in the transitional region (Figure 2a). A high-power view revealed a dense meshwork of GFAP-positive glial processes surrounded by or coinciding with AQP4 immunoreactivity (Figure 2b). AQP4-positive CP epithelial cells lacked GFAP (Figure 2c). Moreover, Figure 2 shows the expansion of subependymal and perivascular astrocytes from the glial plate into the CP stroma. The transition of ependymal cells to CP epithelial cells is indicated by the loss of GFAP.

**Figure 3:**
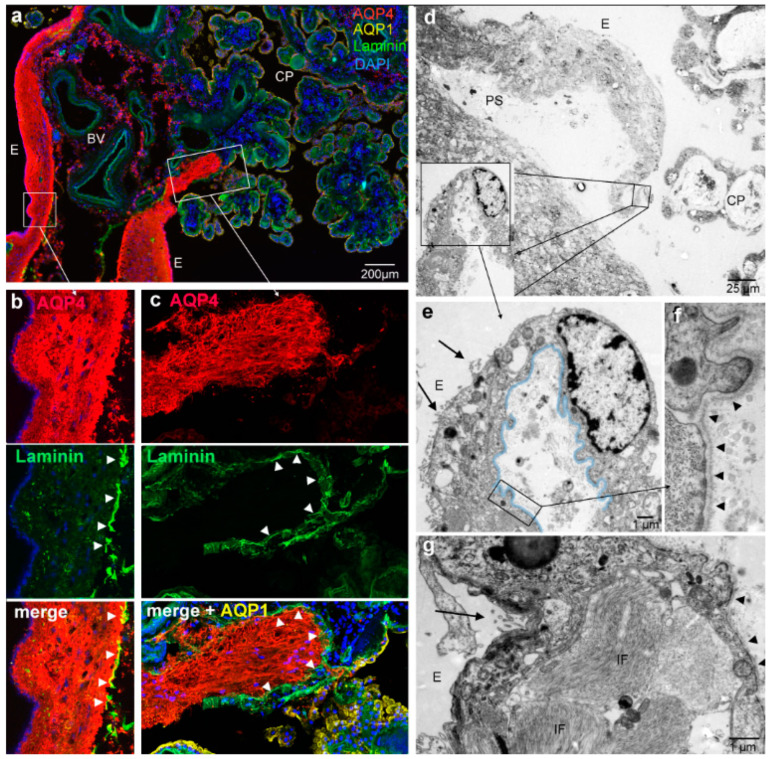
The transitional glial plate is delimited by a basal lamina. (**a**) Immunolabeling for AQP1, AQP4 and laminin reveals a basal lamina under the CP epithelial cells that are apically positive for AQP1. Higher magnification of the indicated areas shows that there is a basal lamina (arrowheads) bordering the astrocytic processes and surrounding blood vessels. (**b**) Moreover, astrocytic processes extending into the stroma (**c**) of the CP are surrounded by laminin staining (**c**, arrowheads). (**d**–**g**) Electron micrographs of the transition zone between ventricle lining ependyma and choroid plexus epithelium. (**d**) An overview of an ependymal/glial surrounding of blood vessels which turns into a single-layered epithelium towards the CP villi on the right. (**e**) Higher magnification of the boxed areas as indicated shows a continuous basal lamina (highlighted in blue) from the multi-layered ependymal side (left) to the single cell layer (right, cell with nucleus). Ependymal cells have apical microvilli (arrows) and occasional cilia. (**f**) Detailed view of the basal lamina under astrocytic processes (arrowheads). (**g**) Astrocytic processes under ependymal cells (E) show large bundles of intermediate filaments (IF). Large arrows point to microvilli, and arrow heads point to the basal lamina. E, ependymal surface; PS, perivascular space; CP, choroid plexus.

To further define this structural change from the ependyma to the CP, we marked the course of the CP epithelium with laminin. The CP epithelial cells are consistently connected to a basal lamina which disappears at the point where the ependymal cells start. The laminin expression underneath AQP4-positive and AQP4-negative CP epithelial cells did not show a difference, as would be expected from previous reports that suggest that laminin plays a role in the AQP4 distribution on rat astrocytes [16,17]. Interestingly, the glial attachment plate formed by astrocytes was surrounded by laminin staining where their processes extended into the stroma (Figure 3c). Laminin was also present at the border of the glial plate on the side facing blood vessels entering the CP (Figure 3a,b). Thus, we can define a perivascular and an ependymal side of the glial plate (Figure 3b). We confirmed a basal lamina on the perivascular side on the ultrastructural level (Figure 3d–f). The EM analysis of the astrocytic processes also showed large bundles of intermediate filaments (Figure 3g) which corresponded to the heavy GFAP staining shown in Figure 2. These astrocytes represented the subependymal zone, and their perivascular side showed a basal lamina which continues to underlie a single cell layer, as can be seen in Figure 3e, defining the transition to the CP epithelium.

**Figure 4:**
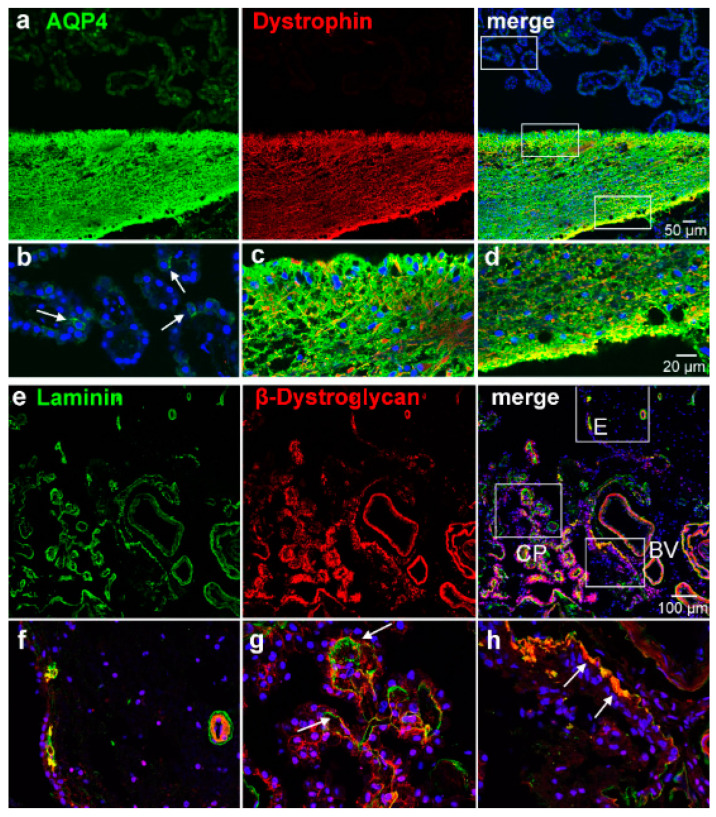
Dystrophin and β-dystroglycan localization in the CP and ependymal transition zone. (**a**) Co-staining with antibodies against AQP4 and dystrophin shows an overlap in the glial subependymal plate but not in the CP. The white rectangles indicate the details shown (**b**–**d**). (**b**) AQP4-positive cells in the CP (arrows) are negative for dystrophin, whereas (**c**,**d**) subependymal astrocytic processes were intensely positive for both dystrophin and AQP4. An even stronger immunoreactivity is found on the perivascular side where the processes face a basal lamina than on the ependymal side of the glial plate (**d**, c.f., Figure 3b,f). (**e**) β-dystroglycan and laminin co-stainings show a close association of both stains. Detailed views (**f**–**h**) of the indicated areas reveal that the ependyma as well as astrocytic processes in the glial plate were immunonegative for β-dystroglycan (**f**), except where they contact a basal lamina as indicated by laminin staining. Both stains are also found in and around blood vessel walls (**h**). In the choroid plexus (CP), epithelial cells express β-dystroglycan basolaterally close to the basal lamina (**g**).

The effect of laminin on AQP4 distribution in astrocytes is thought to be mediated by the dystrophin–glycoprotein complex (DGC). We therefore investigated whether dystroglycan binding to components of the extracellular matrix. such as laminin and dystrophin interacting with the cytoskeleton, corresponded to the expression of AQP4. Indeed, in comparison to astrocytes of the adjacent thalamic brain region (Figure 4a) and similarly to the increased AQP4 expression, the glial plate was heavily immunopositive for dystrophin. However, the AQP4-positive CP epithelial cells were negative for dystrophin (Figure 4b). A higher magnification revealed distinct cellular localizations in astrocytic processes with AQP4 staining often surrounding dystrophin immunoreactivity (Figure 4c). The perivascular side of the glial plate showed an even stronger immunoreactivity for both proteins (Figure 4d). In contrast, the distribution of dystroglycan did not match the pattern of AQP4 we had observed in the glial plate. Instead, it coincided with the immunoreactivity of laminin at blood vessels in the ependyma and on the perivascular side of the glial plate (Figure 4f,h). In addition, CP cells expressed dystroglycan basolaterally where facing the basal lamina delineated by laminin immunoreactivity (Figure 4g). Essentially, all CP epithelial cells were positive for dystroglycan without a correlation to AQP4-expressing cells.

## 4. Discussion

### 4.1. Topography of the Transitional Glial Plate

**Figure 5:**
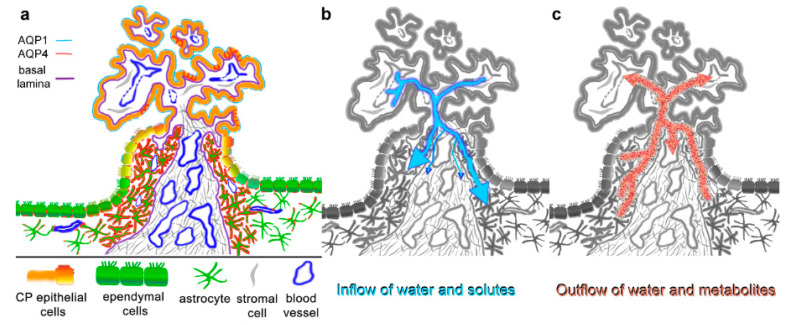
Summary and suggested implications of aquaporin expressions in the ependyma–choroid plexus transition zone. (**a**) AQP4 expression is particularly high in astrocytic processes that form a glial plate or cuff around the blood-supplying vessels of the CP. AQP4 staining is also found basolaterally in some CP epithelial cells (red lines). The astrocytic processes delimited by a basal lamina reach the CP stroma, which includes fenestrated capillaries. AQP1 is expressed mostly apically in CP epithelial cells, which also form tight junctions as the basis for the blood–CSF barrier. Note that the basal lamina of blood vessels is not included in the schematic. (**b**) Water and solutes can diffuse into the CP stroma through fenestrated capillaries and can enter the brain parenchyma via the glial plate. The basal laminae might have a filtering effect but do not constitute a barrier. Water flow is likely restricted by the dense meshwork of astroglial processes in the glial plate. (**c**) Depending on the osmotic gradient, the high density of AQP4 water channels in the glial plate could also serve as a drainage and clearing pathway for metabolites, which are then taken up by postcapillary venules in the stroma. This would contribute significantly to processes suggested by the glymphatic pathway hypothesis.

The investigation of body donor brain samples enabled us to carefully examine the expression of aquaporins in the transition zone between the ependyma and the CP epithelium in the human brain, as well as the border of the subependymal zone to the CP stroma. We showed that the tissue plate surrounding blood vessels entering the CP consists of astrocytes and their processes, which were strongly positive for AQP4. This glial plate is an extension of the subependymal zone and is covered by ependymal cells. The single cell ependymal layer continues on as the CP epithelium; both are derivatives of the neuroepithelial ventricular lining but distinct in their morphology and aquaporin expression [4,18,19] (see Figure 1 and Figure 3). The astrocytic processes in the glial plate form a cuff around the entering blood vessels and can therefore be considered perivascular astrocytic endfeet. On the other hand, the blood vessels are surrounded by leptomeningeal tissues that accompany them into the stroma of the choroid plexus due to developmental processes. Therefore, astrocytic processes in the transition zone can also be considered subpial. In any case, the glial plate is delimited by a basal lamina that is continuous with the basal lamina of the CP epithelium, as illustrated in Figure 5. Our data also showed that astrocytic processes reach and contact the CP stroma in the CP stalk, where they are separated only by the basal lamina.

### 4.2. Astrocytic Polarity in the Transitional Glial Plate

As has been demonstrated many times, perivascular and subpial astroglial endfeet express a high density of AQP4 [20,21], in contrast to their cell somas and perineuronal processes with low or undetectable levels of AQP4. However, the processes in the glial plate and some subependymal areas showed little polarity of an overall high AQP4 expression. The dystrophin–glycoprotein complex (DGC), together with associated proteins such as agrin and alpha-syntrophin, is believed to play a pivotal role in the polarized localization of AQP4 to astrocytic endfeet membranes [22,23,24,25]. The DGC binds to components of the extracellular matrix such as laminin, which recently has been shown to be involved in AQP4 clustering [26]. Additionally, deletion of the associated alpha1-syntrophin leads to an almost complete loss of perivascular AQP4 [27]. Our stainings for beta-dystroglycan and dystrophin showed a differential result of co-localization with AQP4: the unpolarized staining of AQP4 was matched by the distribution of dystrophin in the glial plate but not in the AQP4-positive cells in the CP epithelium. In contrast, beta-dystroglycan was associated with laminin in the glial plate and the CP epithelium, where it was not restricted to AQP4-positive cells. This suggests that the membrane localization of AQP4 is determined by different mechanisms in astrocytic perivascular endfeet, astrocytic processes in the glial plate, and CP epithelial cells. Indeed, AQP4 expression independent of dystrophin has been reported previously for astrocytic subpial endfeet and ependymal cells [28]. For mouse CP cells, the dystrophin homolog utrophin has been localized to the basolateral membrane [29], which might not be recognized by our anti-dystrophin antibody. Thus, variations in the dystrophin–glycoprotein complex might determine local anchoring of AQP4. Since our previous studies in mice suggested an age-related AQP4 expression in the CP epithelium [15], we cannot rule out that the high expression in the glial plate is also due to age-related processes (see below).

### 4.3. Functional Implications of High AQP4 Expression in the Transitional Glial Plate

The blood–brain barrier (BBB) and blood–cerebrospinal fluid barrier (BCSFB) separate the blood milieu from the brain’s internal environment. They are formed by tight junctions located between capillary endothelial cells and between CP epithelial cells, respectively [30]. The capillaries of the CP, however, are fenestrated, allowing for an increased fluid and metabolite exchange, likely to facilitate CSF production [19,31]. The stromal space of the CP between blood vessels and the CP epithelium is not separated from the brain parenchyma by a barrier in the transition zone in the form of tight junctions. Instead, as our data show, it faces a basal lamina and a dense meshwork of AQP4-positive processes.

Previously, the CP has been suggested to be an entry site for immune cells into the brain parenchyma, either by crossing the CP epithelium or by the leptomeningeal route [31,32]. In fact, immune cells, especially macrophages, have been identified in the CP stroma [33]. Parasites such as trypanosomes might enter the brain through the stromal-fenestrated blood vessels as well [34]. Moreover, there is evidence that viruses, including COVID-19, can enter the brain via the CP [35,36]. Likewise, the CP represents an entry site for fluids: as indicated in Figure 5, water and solutes entering the CP stroma from capillaries could flow into the transitional glial plate. Such an outflow has already been pointed out by Brightman and Reese [37], who termed it a ‘functional leak’ and implied the possibility of a bidirectional flow. The dense meshwork of astrocytic processes we observed in the human CP transition zone might form a cuff around CP-entering blood vessels, providing a barrier and limiting stromal fluid from entering the brain interstitial fluid. This might be facilitated by the high level of AQP4 expression on astrocytic processes taking up leaking fluid from the stroma.

It is well documented that CSF production decreases with age and structural changes occur in the CP, such as accumulating deposits and stromal thickening [38,39,40]. Additionally, sex differences in CP gene expression have been reported [41,42]. Thus, age, cause of death, and sex might have an impact on aquaporin expression in the CP and ependyma. However, so far, we have not found any differences in the body donor tissues we analyzed regarding these factors. Along the lines of our previous study [15], it is certainly possible that age has a relevant impact on AQP4 expression in the glial plate, but this remains to be shown.

In the last decade, a recycling and draining fluid system has been proposed for the brain, termed the glymphatic system, recently reviewed in [13]. This system includes the perivascular spaces that connect to the subarachnoid space and serves as a fluid drainage and waste removal system. It is supported by AQP4 in the perivascular endfeet since deletion of AQP4 alters CSF flow [43,44]. Moreover, in AQP4-KO mice the clearing of tau protein CSF and the glymphatic pathway are impaired, and tau accumulates in the brain, causing neurodegenerative symptoms [45]. The high expression of AQP4 in the CP transition plate might be part of the glymphatic drainage system by facilitating water flow and accompanying waste into the CP stroma (Figure 5c).

Since water is driven through aquaporin channels by osmotic and hydrostatic gradients, the direction of water flow through AQP4 in the glial plate cannot be determined with certainty. We propose that the high expression of water channels serves as a control for influx as indicated above, while at the same time or alternatively serving as part of a drainage system that clears into the CP stromal space, where waste products can be taken up by fenestrated blood vessels.

## Figures and Tables

**Table 1:** Primary and secondary antibodies used in this study.

			
Primary AB			
AQP-4	Santa Cruz sc-20812	rabbit	1:100
AQP-4	Santa Cruz sc-9888	goat	1:100
AQP-1	Thermo Fischer PA5-78805	rabbit	1:100
AQP-1	Santa Cruz, sc-32737	mouse	1:100
Dystrophin	abcam ab15277	rabbit	1:100
ß-Dystroglycan	abcam ab49515	mouse	1:100
GFAP	Santa Cruz sc-58766	mouse	1:100
Laminin	abcam ab11575	rabbit	1:100
			
Secondary AB			
Anti-mouse Alexa 488	Thermofisher	goat	1:400
Anti-mouse Alexa 546	Thermofisher	goat	1:400
Anti-rabbit Alexa 488	Thermofisher	goat	1:400
Anti-rabbit Alexa 546	Thermofisher	goat	1:400
Anti-rabbit Alexa 488	Thermofisher	donkey	1:400
Anti-mouse Alexa 546	Thermofisher	donkey	1:400
Anti-goat Alexa 660	Thermofisher	donkey	1:400

## Data Availability

Original image data can be obtained upon reasonable request.

## Supplementary Materials

The following supporting information can be downloaded at: https://www.mdpi.com/article/10.3390/biom13020212/s1, Table S1: Age, sex and postmortem interval of body donors used in this study.
