# Supplementary material for: Aquaporin-1 and Aquaporin-4 Expression in Ependyma, Choroid Plexus and Surrounding Transition Zones in the Human Brain
Source: Biomolecules. 2023 Jan 22;13(2):212. doi: 10.3390/biom13020212 (PMC9953559; doi:10.3390/biom13020212)
Supplement: Supplementary file 1 [file biomolecules-13-00212-s001.zip › biomolecules-2143717-supplementary.pdf]

age	sex	post mortem interval	cause of death*
83 years	female	15 h	pulmonary carcinoma
89 years	male	12 h	kidney failure
91 years	female	19 h	exsiccosis, GI bleeding
75 years	female	8 h	ischemic stroke
74 years	female	9 h	multi organ failure
80 years	female	10 h	cardiac failure
92 years	male	7	acute abdomen

SM Table 1: Age, sex and postmortem interval of body donors used in this study.
* cause of death as stated in the official death certificate after external examination.
